# The Association Between Coworker Support and Work-Family Interference: A Test of Work Environment and Burnout as Mediators

**DOI:** 10.3389/fpsyg.2020.00819

**Published:** 2020-05-05

**Authors:** Leo R. Norling, William J. Chopik

**Affiliations:** Department of Psychology, Michigan State University, East Lansing, MI, United States

**Keywords:** coworker support, work-family interference, mediation, burnout, work environment, turnover

## Abstract

Coworker support has been hypothesized to enhance work-life outcomes. However, the mechanisms underlying this association are unclear. Two studies examined how coworker support predicted work-life outcomes through positive work environment and burnout. It was hypothesized that coworker support enhances work environment, and that better work environment is associated with less burnout; in turn, reduced burnout is associated with less negative work-life interference. In two large studies of working adults (total *N* = 5,666), we found support for our model – coworker support predicted work-family outcomes and this association was mediated by more positive work environments and reduced burnout. Study 2 was a short-term lagged confirmation of the model. Results are discussed in the context of efforts to improve workplace climate, reduce turnover, and improve workers’ job satisfaction.

## Introduction

The integration of work and an individual’s personal life is essential for well-being. With technological advances, employers can now potentially reach employees nearly every hour of every day. Similarly, there are businesses that rely on people being available around the clock. With these increasing demands on employees’ time, people may have insufficient time for both family and work obligations. Although a considerable amount of attention has been dedicated to individual predictors of work-family interference, rarely has research characterized the process through which institutional variables and individual perceptions produce work-family interference (e.g., French et al., 2018). In the current studies, we examine how one predictor – coworker support – is associated with work-family outcomes and the mechanisms that might explain this association. Thus, the current studies consolidate previous work and provide a preliminary test of a sequence through which coworker support contributes to better work environments, lower burnout, and better work-family integration.

### Work-Family Interference

Work life and family life can be stressful for many people. In the modern workplace, workers are tasked with completing their assigned tasks and managing workplace relationships – all of which can be quite depleting, especially in workplaces that are less hospitable (Oyeleye et al., 2013; Ekienabor, 2016). Naturally, family relationships are not without their stressful features either (Sheehan and Nuttall, 1988) – people navigate multiple relationships outside of the work domain that can often put a burden on them, even when those relationships are not inherently conflictual (Rook, 2015; Chopik, 2017). Although both work and family contexts are often examined separately, there are many times in which stress from one domain crosses over and affects the other.

The phenomenon of work-family interference (i.e., stress in one domain affecting functioning in another domain) has been documented in a large number of studies, with both directions – work affecting family and family affecting work – being explored. The study of work-family interference has also been subject to many conceptualizations, coming to describe many adjacent concepts related to the integration of work and other life domains (Wayne et al., 2017; Casper et al., 2018). There is also a number of studies examining the outcomes of poor work environments on work/life outcomes (e.g., Mesmer-Magnus and Viswesvaran, 2005; Ford et al., 2007; Halbesleben, 2010; Amstad et al., 2011).

Family-to-work interference is associated with reduced relationship satisfaction and less satisfaction with household division of labor, especially among women (Stevens et al., 2007). For example, women with young children report higher levels of work-family conflict compared to child free individuals, women with older children, and fathers more generally (Crouter, 1984). Working in a demanding job, a job with little scheduling flexibility, or having little job autonomy are also related to more negative family-to-work interference (Keene and Reynolds, 2005).

Likewise, there is often interference from one’s job that affects their home life. As Ferguson (2012) found, experiences of incivility at work do not solely affect how individuals function in their work environments. Often, they bring it home, where it affects their family relationships, primarily through declines in marital satisfaction (Ferguson, 2012). However, this study did not examine how other factors might alleviate this effect. For instance, meta-analytic research has shown that family support provided by organizations reduces work-family interference (Kossek et al., 2011). Such support may also reduce perceived stressors and time demands, indirectly decreasing work-family conflict (Carlson and Perrewé, 1999). Unfortunately, this meta-analysis did not investigate other variables that might predict reduced work-family interference. For example, coworker support, the quality of the work environment, and burnout are all factors that might predict reduced or enhanced work-family interference (Constable and Russell, 1986; Hamama, 2012; Marek et al., 2017).

### The Contribution of Coworker Support for Work-Family Integration

There are many factors that influence how people evaluate their jobs. For example, the presence of a supportive supervisor and a positive culture are associated with greater productivity and job satisfaction (Lok and Crawford, 1999; Xenikou and Simosi, 2006). To date, research on work environments and work/family conflict has focused mainly on supervisory support, rather than coworker support. Rarely are the factors of coworker support, quality of work environment, and burnout examined simultaneously when predicting outcomes like work-family interference and job satisfaction. Below, we provide a selective review of each of these factors and why they might be associated with work-family interference.

One potential predictor of reduced work-family interference (or even work-family enhancement – in which the conditions of one setting/domain enhance the conditions in another setting/domain) – is coworker support. Coworkers have the potential to enrich an individual’s work experience and their perception of an organization (Ng and Sorensen, 2008). Coworkers can serve many purposes for individuals – they serve as confidants, they help lighten workloads, and they can make difficult work environments more palatable (Neves and Cunha, 2018) – and they ultimately serve as a source of support for individuals. Social support in the workplace significantly contributes to the overall job satisfaction of full-time workers (Ducharme and Martin, 2000), strain reduction (through positive, job-related communication; Beehr et al., 2000) and decreased intentions to quit and turnover in high stress occupations (Ducharme et al., 2007). Additionally, low coworker support, along with other factors (e.g., high job demands, low job control, low procedural and relational justice, and low supervisor support), predicts a higher likelihood of developing a stress-related disorder (e.g., neurasthenia, adjustment disorders, and burnout; Nieuwenhuijsen et al., 2010).

Coworker support can influence one’s perception of the working environment. Specifically, coworker support reduces the harmful effects that unfair treatment by a supervisor has on job satisfaction and psychological distress (Sloan, 2012). That is, coworker support can greatly benefit workers who feel exposed to unfair supervisor treatment.

Another area of ambiguity is why coworker support might be beneficial for work-family interference. Intuitively, it seems that coworker support should make for better work environments (Ducharme and Martin, 2000). It also makes sense that more positive work environments would lead to less burnout (Constable and Russell, 1986) and, in turn, more positive work-family outcomes (Blom et al., 2014). However, the links between all these variables (e.g., coworker support > work environment > burnout > work-family outcomes) are not strongly established. For instance, while one study observed that coworker support reduced burnout (Ducharme et al., 2007), this relationship is not always found (Constable and Russell, 1986). In other words, it is uncertain whether burnout is reduced by coworker support and whether positive coworker interactions create a more positive work environment. Another major contribution of this work is quantifying the degree of directional interference and enhancement. Both directions (work affecting family; family affecting work) are likely to be affected by burnout from either domain. For example, an employee may feel highly stressed from the lack of support they are receiving from their work environment and coworkers. They may find it difficult to manage the cognitive demands in maintaining harmonious work and family lives (Arnsten, 2009). This is especially true among people who feel burnt out from work demands. Being neglectful of family environments because of work demands might also create more family problems which in turn spill-over and negatively affect work. While it is understood that coworker support will affect work to family interaction, we also test the possibility of a bidirectional effect, in which coworker support affects the family to work interactions.

In summary, coworker support may have important effects on one’s work and family life, yet it is unclear to what degree and why. One possibility that we explore is that coworker support enhances work environments. Further, these better work environments may lead to less burnout. Finally, this reduced burnout may lead to less negative work-life interference. In two studies, we tested this possibility – whether the relationship between coworker support and work-family interference is mediated by work environment and burnout.

### The Current Studies

In the current studies, we examined the workplace predictors of work-family interference and enhancement. Specifically, we focused on the quality of coworker relationships and how they are linked with work-family interference. We drew on two samples – one nationally representative survey and one large sample of working adults collected online. We chose two studies to specifically test the boundaries of our process model. In other words, does our model generalize to multiple contexts and developmental settings [for both middle aged and older adults (in Study 1) and younger and middle-aged adults (in Study 2)]? We measured coworker support, work/family interference/enhancement, work environment, and burnout. We tested whether work environment (Study 1) and work environment and burnout (Study 2) mediated the association between coworker support and work/family outcomes.

## Study 1

In Study 1, we sought to establish the link between coworker support and work/family interference/enhancement, and whether this association was mediated by work environment.

### Method

#### Participants

Participants were from the Health and Retirement Study (HRS), a nationally representative prospective panel study that has surveyed more than 22,000 Americans aged 50 and above every 2 years since 1992 (Sonnega et al., 2014). In 2006, a random 50 percent of HRS respondents were given a self-report questionnaire that asked questions about their work environment. The sample comprised of 5,040 older adults (*M*_*age*_ = 60.17, *SD* = 8.19; 56.1% Female; 72% White, 14.7% Black/African American, 9.9% Hispanic, and 3.4% other race/ethnicities, *Mean_*Years Of Education*_* = 13.54, *SD* = 2.77) who completed the survey in either 2010 or 2012 (no participants completed data in both years). Participation was limited to working adults. Participants came from variety of different occupations; the most populous were business operations specialists (21.8%), computer/math occupations (18.5%), management occupations (14.1%), and financial specialists (11.1%). Participants worked an average of 36.86 (*SD* = 13.95) hours per week and have been at their current jobs an average of 12.48 years (*SD* = 11.50).

Because we analyzed an existing data source, the Michigan State Institutional Review Board considered this research exempt from ethical oversight as it did not constitute human subjects research (IRB# 17-1113).

#### Measures^1^

##### Coworker support

Coworker support was assessed with the three-item HRS Coworker Support measure, on a scale ranging from 1 (*strongly disagree*) to 4 (*strongly agree*) (Haynes et al., 1999). A sample item is, “My coworkers listen to me when I need to talk about work-related problems.” Responses were averaged to yield an overall index of coworker support (α = 0.90)^2^.

##### Work environment

Work environment was evaluated with the five-item HRS Work Environment measure, on a scale ranging from 1 (*strongly disagree*) to 4 (*strongly agree*). These items are taken from the 2002 General Social Survey, conducted by the National Opinion Research Center (Smith et al., 2017). A sample item used is, “I have the training opportunities I need to perform my job safely and competently.” Items were averaged to create an overall index of work environment (α = 0.70).

##### Work family interference and enhancement

Work-family exchanges were measured via four scales of the HRS Work/Non-work Interference and Enhancement measure, on a scale ranging from 1 (*rarely*) to 4 (*most of the time*). Interference and enhancement from work to family and family to work was captured by the four scales (MacDermid et al., 2000). The first scale, work interference with family (α = 0.75), was measured with three items (sample item: “Job worries or problems distract me when I am not at work”). The second scale, family interference with work (α = 0.74), was measured with three items (sample item: “I am preoccupied with personal responsibilities while I am at work”). The third scale, work enhancement of family life (α = 0.78), was measured with three times (sample item: “My work gives me energy to do things with my family and other important people in my life”). Lastly, the fourth scale, family life enhancement of work (α = 0.84), was measured with three items as well (sample item: “I am in a better mood at work because of my family or personal life”).

##### Job satisfaction

Job satisfaction was measured with a nine-item HRS Job Satisfaction scale ranging from 1 (*strongly disagree*) to 4 (*strongly agree*) (Karasek, 1979; Smith et al., 2017). A sample item is, “All things considered I am satisfied with my job.” Responses were averaged to yield an overall index of coworker support (α = 0.82).

#### Analytic Approach

For Study 1, we first began by examining the study descriptives and bivariate correlations between the main study variables. In order to further isolate the effect of coworker support on the mediator (i.e., workplace environment) and each outcome, we ran linear regressions predicting workplace environment, work/family interference/enhancement, and job satisfaction from coworker support, controlling for age, gender, education, hours worked per week, and tenure. Based on the results of these regression analyses (i.e., that the variables were associated in ways that we thought), we proceeded to test our hypothesized mediation model. To test whether work environment mediated the association between coworker support and work-family outcomes, we used Hayes (2013) PROCESS macro (i.e., Model 4)^3^. These mediation analyses tested the significance of the indirect effect and whether the direct association between an independent variable and a dependent variable is significantly reduced after the inclusion of an explanatory (i.e., mediating) variable. This analysis provides point estimates and confidence intervals for the indirect effect of a mediator. Confidence intervals are derived through bootstrapping (with 5,000 bootstrapped samples). Coworker support (X) was entered as a predictor of each work-family outcome and job satisfaction separately (Y) mediated through work environment (M), controlling for age, gender, education, work hours, and tenure. Finally, we examined whether these associations were robust to a number of controls.

### Results and Discussion

#### Preliminary Analyses

Sample descriptives and correlations between variables can be found in Table 1. At the bivariate level, coworker support was associated with a more positive work environment and less work/family life conflict and more work/family life enhancement. Men reported a more positive working environment. Older adults reported greater coworker support, a more positive working environment, less conflict, and more enhancement. Older adults typically exhibit a “positivity bias” for evaluative ratings, particularly of their social circumstances and these findings are consistent with that literature (Reed and Carstensen, 2012). People with more education report more coworker support and a more positive working environment. People who work more hours a week report a less positive work environment, more work/life conflict, and less work/life enhancement. The number of work hours often serves as a proxy measure for work-related stress (because it portends fewer breaks and more intense work periods); these results are consistent with work hours predicting signs of employee burnout in previous research (Linzer et al., 2001). Work environment and the conflict/enhancement measures were all correlated in intuitive directions.

**TABLE 1:** Correlations and descriptives for all study variables for Study 1.

	*M*	*SD*	1	2	3	4	5	6	7	8	9	10	11
(1) Gender	–	–	–										
(2) Age	60.17	8.19	−0.15**	–									
(3) Education	13.54	2.77	–0.03	−0.03*	–								
(4) Hours/week	36.86	13.95	−0.17**	−0.28**	0.06**	–							
(5) Tenure	12.41	11.5	−0.12**	0.08**	0.05**	0.17**	–						
(6) Coworker support	3.2	0.62	–0.02	0.08**	0.16**	–0.02	0.03*	–					
(7) Work environment	3.05	0.54	−0.08**	0.18**	0.12**	−0.10**	0.04*	0.61**	–				
(8) Work interfering w/family	1.56	0.57	0.004	−0.16**	0.02	0.29**	0.03*	−0.23**	−0.34**	–			
(9) Family interfering w/Work	1.17	0.34	0.01	−0.67**	−0.05**	0.04**	0.01	−0.15**	−0.18**	0.36**	–		
(10) Work enhancing family	2.81	0.88	–0.03	0.19**	0.04**	−0.22**	−0.04**	0.27**	0.41**	−0.52**	−0.22**	–	
(11) Family enhancing work	3.21	0.8	−0.03**	0.10**	0.06**	−0.06**	0.004	0.25**	0.31**	−0.33**	−0.34**	0.63**	–
(12) Job satisfaction	2.94	0.52	−0.07**	0.18**	0.11**	−0.07**	0.03**	0.49**	0.67**	−0.36**	−0.19**	0.44**	0.32**

Gender: 1 = male, 2 = female. Education: Years of Education. Tenure: Years are Current Job. *p < 0.05, **p < 0.01.

#### Regression Analyses

The results of the regression analyses can be found in Table 2. Across all outcomes (work environment and work-family interference/enhancement, job satisfaction), coworker support was associated with a more positive work environment, less work-life conflict, more work-life enhancement, and more job satisfaction.

**TABLE 2:** Regressions predicting work environment, work-family interference, and work-family enhancement.

	*B*	*SE*	β	*t*	*p*	95% CI	
**Work environment**							
Intercept	1.03	0.08		13.10	<0.001	LB	UB
Coworker support	0.52	0.01	0.60	49.94	<0.001	0.50	0.54
Gender	−0.06	0.01	−0.05	−4.25	<0.001	−0.08	−0.03
Age	0.01	0.001	0.11	8.52	<0.001	0.01	0.01
Education	0.004	0.002	0.02	1.52	0.13	−0.001	0.01
Hours/week	−0.002	0.001	−0.05	−3.67	<0.001	−0.003	−0.001
Tenure	<0.001	0.001	0.01	0.82	0.42	−0.001	0.002
**Work interfering w/family**							
Intercept	1.78	0.10		17.33	<0.001	LB	UB
Coworker support	−0.21	0.01	−0.22	−15.17	<0.001	−0.23	−0.18
Gender	0.04	0.02	0.04	2.43	0.02	0.01	0.08
Age	−0.003	0.001	−0.05	−2.94	0.003	−0.01	−0.001
Education	0.01	0.003	0.06	3.78	<0.001	0.01	0.02
Hours/week	0.01	0.001	0.27	17.24	<0.001	0.01	0.01
Tenure	−0.001	0.001	−0.01	−0.68	0.50	−0.002	0.001
**Family interfering w/work**							
Intercept	1.5	0.06		24.36	<0.001	LB	UB
Coworker support	−0.07	0.01	−0.13	−8.16	<0.001	−0.08	−0.05
Gender	0.01	0.01	0.01	0.55	0.58	−0.02	0.03
Age	−0.002	0.001	−0.06	−3.52	<0.001	−0.004	−0.001
Education	−0.002	0.002	−0.02	−1.27	0.20	−0.01	0.001
Hours/week	0.001	<0.001	0.03	1.74	0.08	<0.001	0.001
Tenure	<0.001	<0.001	0.02	1.02	0.31	<0.001	0.001
**Work enhancing family**							
Intercept	1.34	0.16		8.58	<0.001	LB	UB
Coworker support	0.35	0.02	0.25	17.12	<0.001	0.31	0.39
Gender	−0.05	0.03	−0.03	−2	0.05	−0.10	−0.001
Age	0.01	0.002	0.12	8.02	<0.001	0.01	0.02
Education	0.002	0.01	0.01	0.42	0.67	−0.01	0.01
Hours/week	−0.01	0.001	−0.17	−11.10	<0.001	−0.01	−0.01
Tenure	−0.001	0.001	−0.02	−1.19	0.23	−0.004	0.001
**Family enhancing work**							
Intercept	1.78	0.15		12.13	<0.001	LB	UB
Coworker support	0.30	0.02	0.24	15.65	<0.001	0.26	0.34
Gender	−0.01	0.02	−0.01	−0.31	0.75	−0.06	0.04
Age	0.01	0.002	0.07	4.69	<0.001	0.004	0.01
Education	0.01	0.004	0.03	1.93	0.05	<0.001	0.02
Hours/week	−0.003	0.001	−0.04	−2.63	0.01	−0.004	−0.001
Tenure	< 0.001	0.001	0.002	0.13	0.90	−0.002	0.002
**Job satisfaction**							
Intercept	1.15	0.08		13.76	<0.001	LB	UB
Coworker support	0.39	0.01	0.47	35.39	<0.001	0.37	0.41
Gender	−0.03	0.01	−0.03	−2.18	0.03	−0.06	−0.003
Age	0.01	0.001	0.13	9.04	<0.001	0.01	0.01
Education	0.01	0.003	0.03	2.34	0.02	0.001	0.01
Hours/week	−0.001	0.001	−0.02	−1.20	0.23	−0.002	< 0.001
Tenure	0.001	0.001	0.01	1.00	0.32	−0.001	0.002

#### Mediation Analysis

Because coworker support predicted work-family and job satisfaction outcomes (the “c” path) and work environment (the “a” path), we examined whether the relationship between coworker support and work-family-job outcomes was explained (i.e., mediated) by work environment. Because work environment was a significant predictor of work-family outcomes and job satisfaction (the “b” path), we were justified in using this particular mediation model.

**FIGURE 1:**
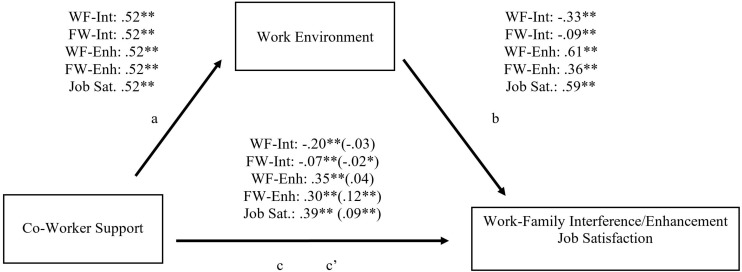
A mediational model of coworker support predicting work/family outcomes through work environment. W, Work, F, Family, Int, Interference, Enh, Enhancement, Sat, Satisfaction. *p < 0.05, **p < 0.01.

As seen in Figure 1, this mediation model suggested that more coworker support was associated with a more positive work environment, which in turn was associated with better work-family outcomes (e.g., lower interference and more enhancement) and greater job satisfaction. The initial associations between coworker support and work-family-job outcomes were reduced after considering work environment as a mediator (often to non-significance). These findings provide support for our proposed model; specifically, coworker support contributes to a more positive workplace environment. This positive workplace environment provides many benefits to workers, including better work/family integration and greater job satisfaction. Significant mediation was confirmed with significant point estimates for each model (see Table 3).

**TABLE 3:** Indirect effect point estimates for Figure 1.

	Y = WF Int	Y = FW Int	Y = WF Enh	Y = FW Enh	Y = Job satisfaction
Effect	–0.17	–0.05	0.31	0.19	0.30
Boot SE	0.01	0.01	0.02	0.02	0.01
BootLLCI	–0.20	–0.06	0.28	0.16	0.28
BootULCI	–0.15	–0.03	0.35	0.22	0.33

*Boot SE, Bootstrapped standard error; BootLLCI, Bootstrapped lower confidence interval; BootULCI, Bootstrapped upper confidence interval. Effects with confidence intervals that do not include zero are statistically significant.*

#### Supplementary Analyses

Due to the correlational nature of the study, there is a possibility that some of the associations between coworker support, work environment, and each outcome might be attributable to the influence other variables, including characteristics of the individual and their workplace. For example, variation in work/family interference and enhancement might be attributable to their personality, their relationship with their supervisor, experiences of discrimination at work, or their mental health. To address this, we took a broad approach and further integrated ostensibly related variables from the HRS. Specifically, we re-ran the analyses reported above while also controlling for Big Five personality traits (John and Srivastava, 1999), depression (Radloff, 1977), workplace discrimination (McNeilly et al., 1996; Xu and Chopik, 2020), perceived control (Pearlin and Schooler, 1978), optimism (Scheier et al., 1994; Chopik et al., 2015), and supervisor support (Haynes et al., 1999). These analyses can be seen in Supplementary Table S8. In every scenario, coworker support was a robust and significant predictor of each outcome (βs > |0.04|, *p*s < 0.03). Mediation analyses were also re-run (see Supplementary Table S9). Of the five outcomes, workplace environment still mediated the association between coworker support and the outcomes (Estimates > |0.05|, *SE*s = 0.01, 95% CI closest to zero: |0.04| to |0.06|). The one exception was the indirect effect for family interference with work, although even this effect was marginally significant and in the predicted direction, *p* = 0.08. Thus, to the extent that these personal and job characteristics affect the mediator and outcomes presented here, coworker support can be considered an independent predictor of each outcome.

## Study 2

**FIGURE 2:**
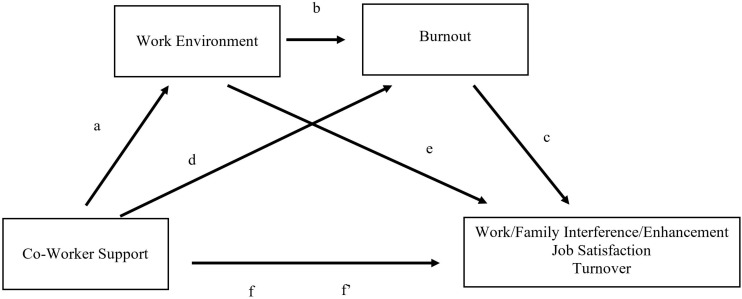
A serial mediation model of coworker support predicting work/family outcomes through work environment and burnout.

For Study 2, we examined why a better work environment resulting from greater coworker support leads to better work-family outcomes. Burnout is one such mechanism that may explain this association. We collected a new sample and tried to replicate the associations found in Study 1 and added burnout as an additional mediator in a serial mediation model (see Figure 2). We hypothesized that coworker support would lead to a more positive work environment, which would lead to lower burnout, which would lead to better work-family outcomes. We tested this hypothesis through a short-term lagged study (e.g., two separate surveys, taken 8 weeks apart)^4^.

### Method

#### Participants

Mechanical Turk was used to collect participant data. Participants took an initial survey that asked questions about coworker support and work environment. Of the Time 1 participants, 626 participants completed a follow-up survey 8-weeks later that asked questions regarding burnout and work-family outcomes. Participants were paid $0.50 for completing each survey (i.e., $1.00 total).

We surveyed 1,062 participants (*M*_*age*_ = 36.18, *SD* = 11.17; 57.6% Female; 76% White, 9% Black/African American, 7% Hispanic, 6% Asian, and 3% other race/ethnicities; 48.1% College Degree or Equivalent (e.g., Bachelors, Associates) and restricted participation to working adults with full-time jobs (*M_*Hours worked per week*_* = 36.89, *SD* = 9.64), with an 85% approval rating on Mechanical Turk. Fifty-three percent of participants had children and reported various marital statuses (31% single, 15% living with partner, 44% married, 8% divorced, and 2% widowed). Participants came from a wide variety of job occupations. The most frequent occupations were business operations (18%), management occupations (17%), computer/math occupations (15%), financial specialists (9%), and education (6%). Participants worked an average of 36.89 (*SD* = 9.64) hours per week and have been at their current jobs an average of 4.42 years (*SD* = 4.66).

Those who did complete Time 2 were comparable to those who did not on all study variables of interest (*p*s > 0.71). Compared to participants with complete data, those who dropped out were younger (*d* = 0.25), less educated (*d* = 0.14), and had less tenure at their current jobs (*d* = 0.15).

This study was carried out in accordance with the recommendations of Michigan State University Institutional Review Board with informed consent being secured from all subjects. The study was conducted online; documentation of written consent was waived by the MSU Institutional Review Board, albeit informed consent was still secured in the online survey. All subjects gave informed consent in accordance with the Declaration of Helsinki. The protocol was approved by the Michigan State University Institutional Review Board (MSU IRB# x17-1182e).

#### Measures

The materials were largely the same as Study 1, with some exceptions. Specifically, all the measures from the HRS were included in this second sample. However, we expanded the measurement of these constructs by including additional scales to measure burnout, job satisfaction, and turnover.

##### Time 1

Participants completed an initial survey of demographics, coworker support, and work environment.

###### Coworker support

Measures included the same three items used in study 1, with the addition of six more items (nine items total) from the broader Coworker Support Scale developed by Caplan et al. (1975). The scale was also adapted to be a seven-point Likert scale, ranging from 1 (*strongly disagree*) to 7 (*strongly agree*). An example item used is, “My coworkers go out of their way to make my life easier.” Responses were averaged to create an overall composite of coworker support (α = 0.95; Caplan et al., 1975).

###### Work environment

The Survey of Perceived Organizational Support was used as a measure of work environment in Study 2. Participants evaluated eight items on a scale ranging from 1 (*strongly disagree*) to 7 (*strongly agree*). A sample item used is, “My organization would forgive an honest mistake on my part.” Responses were averaged to create an overall composite of work environment (α = 0.90; Eisenberger et al., 1997).

##### Time 2

Participants completed a follow-up survey measuring the outcome variables: work-family outcomes, burnout, job satisfaction, and turnover.

###### Work family interference and enhancement

Work-family exchanges were measured utilizing the same four scales from the HRS, used previously in Study 1: work interference with family (α = 0.81); family interference with work (α = 0.82); work enhancement of family (α = 0.78); family enhancement of work (α = 0.79).

###### Burnout

Burnout was measured through a 14-item Shirom-Melamed Burnout Measure (SMBM; Shirom, 2005, 2011) on a seven-point scale, ranging from 1 (*never or almost never*) to 7 (*always or almost always*). The questions were asked in reference to how often one felt in the past 30 workdays. Sample items include “I feel physically drained,” “I feel I am unable to be sensitive to the needs of coworkers and customers,” and “I feel like I am not thinking clearly.” Responses were averaged to create an index of burnout (α = 0.96).

###### Job satisfaction

Job satisfaction was assessed with the same measure from Study 1. Responses were averaged to create an overall index of job satisfaction (α = 0.85).

###### Turnover

Turnover was evaluated with the 3-item Turnover Measure developed by Cook et al. (1981). Participants responded to three items (e.g., “I will actively look for a new job in the next year”) on a 5-point scale, ranging from 1 (*strongly disagree*) to 5 (*strongly agree*) (Cook et al., 1981). Responses were averaged to create an index of turnover (α = 0.92).

#### Analytic Approach

Like Study 1, we began by examining the study descriptives and bivariate correlations between the main study variables. In order to further isolate the effect of coworker support on the mediators (i.e., workplace environment, burnout) and each outcome, we ran linear regressions predicting workplace environment, burnout, work/family interference/enhancement, job satisfaction, and turnover from coworker support, controlling for age, gender, education, hours worked per week, and tenure. Based on the results of these regression analyses (i.e., that the variables were associated in ways that we thought), we proceeded to test our hypothesized mediation model. To test whether work environment mediated the association between coworker support and work-family outcomes, we used Hayes (2013) PROCESS macro (i.e., Model 6). These mediation analyses tested the significance of the indirect effect and whether the direct association between an independent variable and a dependent variable is significantly reduced after the inclusion of explanatory (i.e., mediating) variables. Coworker support (X) was entered as a predictor of each outcome separately (Y) mediated through work environment (M1) and burnout (M2), controlling for age, gender, education, work hours, and tenure. Finally, we examined whether these associations were robust to a number of controls.

### Results and Discussion

#### Preliminary Analyses

Sample descriptives and correlations between variables can be found in Table 4. The vast majority of associations replicated Study 1. The few that did not involve demographic differences in work environment and associations between hours worked and conflict/enhancement. Older adults and people with greater tenure felt less burnt out and lower intentions to leave their job (i.e., turnover). People working more hours also reported lower intentions to leave their job. Higher levels of coworker support were associated with less burnout, higher job satisfaction, and lower turnover. Less conflict and more enhancement (for work/family life) were each associated with less burnout, higher job satisfaction, and lower turnover.

**TABLE 4:** Correlations and descriptives for all study variables in Study 2.

	*M*	*SD*	1	2	3	4	5	6	7	8	9	10	11	12	13
(1) Gender	–	–	–												
(2) Age	36.18	11.17	−0.07*	–											
(3) Education	3.95	1.1	0.01	0.09**	–										
(4) Hours/week	36.89	9.64	0.12*	0.08*	0.10**	–									
(5) Tenure	4.42	4.66	–0.06	0.47**	0.06	0.09**	–								
(6) Coworker support	5.05	1.14	–0.02	0.09**	0.08*	0.08*	0.10**	–							
(7) Work environment	3.66	0.79	0.01	0.06	0.01	0.02	0.09**	0.64**	–						
(8) Work interfering w/family	1.77	0.7	0.003	−0.09*	–0.02	0.04	–0.05	−0.28**	−0.32**	–					
(9) Family interfering w/work	1.53	0.63	0.15**	−0.19**	–0.04	0.05	0.001	−0.18**	−0.16**	0.59**	–				
(10) Work enhancing family	2.41	0.8	0.08	0.08*	0.04	–0.05	0.15**	0.36**	0.44**	−0.40**	−0.18**	–			
(11) Family enhancing work	2.74	0.77	−0.09*	0.17**	0.05	–0.001	0.14**	0.32**	0.26**	−0.36**	−0.42**	0.59**	–		
(12) Burnout	3.13	1.26	–0.07	−0.19**	–0.07	0.02	−0.13**	−0.41**	−0.41**	0.58**	0.49**	−0.48**	0.48**	–	
(13) Job satisfaction	2.71	0.53	–0.01	0.01	0.06	0.04	0.10*	0.52**	0.65**	−0.40**	−0.21**	0.58**	0.42**	−0.57**	–
(14) Turnover	2.66	1.29	–0.02	−0.14**	0.003	−0.11**	−0.16**	−0.37**	−0.49**	0.38**	0.25**	−0.42**	−0.30**	0.47**	−0.65**

Gender: 1 = male, 2 = female. Education: Years of Education. Tenure: Years are Current Job. * p < 0.05, ** p < 0.01.

#### Regression Analyses

The results of the regression analyses can be found in Table 5. Across all outcomes, coworker support was associated with a more positive work environments, less work-life conflict, more work-life enhancement, lower burnout, higher job satisfaction, and lower turnover intentions.

**TABLE 5:** Regressions predicting work environment, work-family interference, work-family enhancement, burnout, job satisfaction, and turnover in Study 2.

	*B*	*SE*	β	*t*	*p*	95% CI	
**Work environment**							
Intercept	1.52	0.15		10.05	<0.001	LB	UB
Coworker support	0.45	0.02	0.65	24.99	<0.001	0.41	0.48
Gender	0.05	0.04	0.03	1.10	0.27	−0.04	0.13
Age	<0.001	0.002	−0.004	−0.14	0.89	−0.004	0.004
Education	−0.02	0.02	−0.03	−1.29	0.20	−0.06	0.01
Hours/week	−0.003	0.002	−0.03	−1.21	0.23	−0.01	0.002
Tenure	0.01	0.01	0.03	1.03	0.30	−0.01	0.02
**Work interfering w/family**							
Intercept	2.70	0.21		12.70	<0.001	LB	UB
Coworker support	−0.18	0.03	−0.29	−7.05	<0.001	−0.23	−0.13
Gender	−0.02	0.06	−0.02	−0.37	0.71	−0.14	0.09
Age	−0.01	0.003	−0.07	−1.62	0.11	−0.01	0.001
Education	0.001	0.03	0.002	0.05	0.96	−0.05	0.05
Hours/week	0.004	0.003	0.05	1.22	0.22	−0.002	0.01
Tenure	<0.001	0.001	−0.002	−0.03	0.97	−0.01	0.01
**Family interfering w/work**							
Intercept	2.14	0.19		11.20	<0.001	LB	UB
Coworker support	−0.10	0.02	−0.17	−4.28	<0.001	−0.14	−0.05
Gender	0.17	0.05	0.14	3.26	0.001	0.07	0.28
Age	−0.01	0.003	−0.23	−5.09	<0.001	−0.02	−0.01
Education	−0.01	0.02	−0.02	−0.46	0.65	−0.06	0.04
Hours/week	0.002	0.003	0.03	0.68	0.50	−0.004	0.01
Tenure	0.02	0.01	0.13	2.82	0.01	0.01	0.03
**Work enhancing family**							
Intercept	1.04	0.24		4.44	<0.001	LB	UB
Coworker support	0.25	0.03	0.36	9.16	<0.001	0.20	0.31
Gender	0.18	0.07	0.11	2.69	0.01	0.05	0.30
Age	<0.001	0.003	0.01	0.12	0.91	−0.01	0.01
Education	0.01	0.03	0.02	0.41	0.68	−0.05	0.07
Hours/week	−0.01	0.004	−0.10	−2.49	0.01	−0.02	−0.002
Tenure	0.02	0.01	0.14	3.11	0.002	0.01	0.04
**Family enhancing work**							
Intercept	1.54	0.23		6.69	<0.001	LB	UB
Coworker support	0.20	0.03	0.30	7.52	<0.001	0.15	0.26
Gender	−0.13	0.06	−0.08	−2.01	0.05	−0.25	−0.003
Age	0.01	0.003	0.11	2.53	0.01	0.002	0.01
Education	0.01	0.03	0.01	0.34	0.74	−0.05	0.07
Hours/week	<0.001	0.003	−0.01	−0.13	0.89	−0.01	0.01
Tenure	0.01	0.01	0.06	1.38	0.17	−0.004	0.03
**Burnout**							
Intercept	6.11	0.36		17.12	<0.001	LB	UB
Coworker support	−0.44	0.04	−0.40	−10.39	<0.001	−0.52	−0.35
Gender	−0.24	0.10	−0.09	−2.40	0.02	−0.43	−0.04
Age	−0.02	0.01	−0.16	−3.61	<0.001	−0.03	−0.01
Education	−0.03	0.04	−0.03	−0.76	0.45	−0.12	0.05
Hours/week	0.01	0.01	0.07	1.87	0.06	−0.001	0.02
Tenure	−0.01	0.01	−0.04	−0.91	0.36	−0.03	0.01
**Job satisfaction**							
Intercept	1.40	0.14		9.7	<0.001	LB	UB
Coworker support	0.24	0.02	0.52	14.47	<0.001	0.21	0.28
Gender	0.08	0.04	0.08	2.06	0.04	0.004	0.16
Age	−0.004	0.002	−0.08	−1.92	0.06	−0.01	<0.001
Education	0.01	0.02	0.02	0.46	0.65	−0.03	0.04
Hours/week	<0.001	0.002	0.003	0.07	0.94	−0.004	0.004
Tenure	0.01	0.01	0.10	2.51	0.01	0.003	0.02
**Turnover**							
Intercept	5.41	0.38		14.37	<0.001	LB	UB
Coworker support	−0.41	0.04	−0.36	−9.31	<0.001	−0.50	−0.32
Gender	−0.04	0.10	−0.02	−0.37	0.71	−24	0.17
Age	−0.01	0.01	−0.06	−1.27	0.20	−0.02	0.003
Education	0.07	0.05	0.06	1.43	0.15	−0.03	−0.002
Hours/week	−0.01	0.003	−0.09	−2.34	0.02	−0.03	−0.002
Tenure	−0.03	0.01	−0.11	−2.47	0.01	−0.06	−0.01

#### Mediation Analysis

The mediation analysis for Study 2 is presented in Figure 2. Because coworker support predicted each outcome (the “f” path), work environment (the “a” path), and burnout (the “d” path), we examined whether the relationship between coworker support and each outcome was explained (i.e., mediated) by work environment and burnout. Specifically, we hypothesized that greater coworker support would be associated with a better work environment, which in turn would be associated with lower burnout. Lower burnout would then in turn be associated with less work/family interference, greater work/family enhancement, greater job satisfaction, and less turnover.

Path estimates can be found in Table 6. As seen in Figure 2, this mediation model suggested that more coworker support was associated with a more positive work environment, which in turn was associated with less burnout, which in turn was associated with better work-family outcomes (e.g., lower interference and more enhancement), greater job satisfaction, and lower turnover. The initial associations between coworker support and work-family outcomes were reduced after considering work environment and burnout as mediators (occasionally to non-significance). Significant mediation (and mediation for the subordinate serial components) was confirmed with significant point estimates for each model (see Table 7). The only exception to this pattern is non-significant family to work influences for both enhancement and interference [i.e., coworker support predicting family-work outcomes through work environment (paths a/e)]. This suggests that coworker support might not affect the transference of stress/enhancement from family to work through changes to the work environment. This lack of finding makes conceptual sense – coworker support primarily affects how experiences at work spill-over to home (and not vice versa).

**TABLE 6:** Path Estimates for Figure 2.

Path	*a*	*b*	*c*	*d*	*e*	*f*	*f’*
Work-family interference	0.47**	−0.36**	0.31**	−0.27**	−0.09*	−0.18**	–0.001
Family-work interference	0.47**	−0.36**	0.26**	−0.27**	0.05	−0.10**	–0.004
Work-family enhancement	0.47**	−0.36**	−0.22**	−0.27**	0.28**	0.25**	0.03
Family-work enhancement	0.47**	−0.36**	−0.25**	−0.27**	–0.01	0.20**	0.10*
Job satisfaction	0.47**	−0.36**	−0.16**	−0.27**	0.30**	0.24**	0.04*
Turnover	0.47**	−0.36**	0.33**	−0.27**	−0.57**	−0.41**	0.002

* p < 0.05, ** p < 0.01.

**TABLE 7:** Indirect effect point estimates for Figure 2.

Indirect effects of X on Y	Y = WF Int	Y = FW Int	Y = WF Enh	Y = FW Enh	Y = Job satisfaction	Y = Turnover
Ind1 Effect	–0.04	0.02	0.13	–0.003	0.14	–0.27
Ind1 Boot SE	0.02	0.02	0.02	0.03	0.02	0.05
Ind1 BootLLCI	–0.09	–0.01	0.09	–0.05	0.10	–0.37
Ind1 BootULCI	–0.001	0.06	0.18	0.05	0.18	–0.17
Ind2 Effect	–0.05	–0.04	0.04	0.04	0.03	–0.06
Ind2 Boot SE	0.01	0.01	0.01	0.01	0.01	0.02
Ind2 BootLLCI	–0.08	–0.07	0.02	0.02	0.01	–0.09
Ind2 BootULCI	–0.03	–0.02	0.06	0.07	0.04	–0.03
Ind3 Effect	–0.08	–0.07	0.06	0.07	0.04	–0.09
Ind3 Boot SE	0.02	0.02	0.02	0.02	0.01	0.02
Ind3 BootLLCI	–0.12	–0.11	0.03	0.04	0.02	–0.14
Ind3 BootULCI	–0.05	–0.04	0.09	0.10	0.07	–0.05

W, Work; F, Family; Int, Interference; Enh, Enhancement; Boot SE, standard error; BootLLCI, Bootstrapped lower confidence interval; BootULCI, Bootstrapped upper confidence interval. Ind1 = a* e. Ind2 = a* b^∗^c. Ind3 = d* c (see Figure 2). Effects with confidence intervals that do not include zero are statistically significant.

#### Supplementary Analyses

Like Study 1, Study 2 was correlational in nature and likewise leaves open the possibility that the associations observed might be attributable to the influence of other variables. Because Study 2 was conceived of as a replication and extension of Study 1 (and was not as large a data collection effort as HRS), fewer variables were available to isolate these associations. Nevertheless, some data were available to reduce the possibility that the links between coworker support, work environment, and burnout, and all the outcomes. Specifically, we additionally collected data on supervisor support (Haynes et al., 1999) and job stress (e.g., “I am under constant time pressure due to a heavy workload”; Karasek, 1979). We re-ran the analyses reported above while controlling for these two variables (see Supplementary Table S10). In every scenario, coworker support was a robust and significant predictor of each outcome (βs > |0.11|, *p*s < 0.01). Mediation analyses were also re-run (see Supplementary Table S11). For the six outcomes, workplace environment and burnout still serially mediated the association between coworker support and the outcomes (Estimates > |0.03|, *SE*s < 0.02, 95% CI closest to zero: |0.01| to |0.04|). Thus, to the extent that supervisor support and job stress affects the mediators and outcomes presented here, coworker support can be considered an independent predictor of each outcome.

## General Discussion

The current studies evaluated the extent to which coworker support predicted work-family interference/enhancement, job satisfaction, and turnover. The studies also focused on how work environment and burnout may explain the associations between coworker support and each work/family outcome. Across the two studies, we found that coworker support was associated with a more positive work environment, less work-life conflict, more work-life enhancement, lower burnout, higher job satisfaction, and lower turnover intentions. More positive work environments and reduced burnout mediated the associations between coworker support and each outcome for the majority of cases.

The fact that work environment and burnout mediated the association between coworker support and work-family interference/enhancement, job satisfaction, and turnover is important for many reasons. Identifying the process through which coworker support affects work-related outcomes also enables organizations to make realistic assessments for how to improve the lives of their coworkers. For example, organizations could provide more opportunities for coworkers to socialize and develop support systems among each other. These opportunities could take the form of team building exercises, employee celebrations, or even lunch-hour cook-outs (Naylor et al., 1996; Staggers et al., 2008; Taylor, 2012). An important direction for future research is to take into account the constellation of factors that contribute to worker well-being and support. For example, a burgeoning literature not explicitly represented in the current report is how workplace leadership (Eisenberger et al., 2002), organizational culture, and management policies (Thompson and Prottas, 2006) also affect downstream work-family-job outcomes. Due to these factors often being examined in isolation of one another, a comprehensive examination of how all of these factors jointly and simultaneously contribute to work outcomes for employees is needed.

The results from the current studies align well with previous research examining coworker support and work-family outcomes. In particular, it appears that coworker support predicts a better work environment (Ducharme and Martin, 2000). Intuitively, more positive work environments would lead to less burnout (Constable and Russell, 1986), and therefore, more positive work-family outcomes (Blom et al., 2014). Nonetheless, there was a gap in the literature as to *why* coworker support might be beneficial for work-family interference. These links between possible constructs (e.g., coworker support > work environment > burnout > work/family outcomes) were not strongly established or explicitly tested before being included in the process models seen in Figures 1, 2. It was also unknown the degree of directional influence between coworker support on interference and enhancement. We found that coworker support affects many aspects of a worker’s life – not only how stress at work translates to the home environment but also how stress at home can create difficulty in work environments. Although our process model was primarily true for work outcomes (e.g., satisfaction, turnover) and work-family interference (but not family-work interference; see Figure 2), the current studies provide a strong test for the ways in which work and family lives are intertwined and how aspects in one domain (coworker support) can alleviate problematic crossovers between the two settings. Overall, the inclusion of work environment and burnout as mediators of the association between coworker support and work-family outcomes has the potential to motivate future research in organizational psychology.

### Strengths, Limitations, and Future Research

The current studies had many strengths. We used two large studies of working adults and modeled many possible variables relevant to work and family life. We examined the influence of coworker support in the sequence that leads to more positive workplace environments and then to downstream outcomes that are important for everyday people. Previous research had provided piecemeal evidence for this process. Coworker support has been shown to enrich workplace environments, positive workplace environments reduce burnout, and positive workplace environments and lower burnout often translate to better outcomes for individuals. But the two studies presented here provide a more detailed test of the sequence through which coworker support affects work outcomes for people. We also expanded this question by simultaneously examining several outcomes, including work/family interference and enhancement, job satisfaction, and job turnover. The fact that coworker support predicted work/family interference, how satisfied people are with their jobs, and whether or not they planned to quit their jobs is a testament to the important role that coworkers play in people’s lives.

The inclusion of a broad set of control variables – with respect to both individual and job characteristics – allowed us to further isolate the effect that coworker support has on work/family outcomes. That personal (e.g., personality, depression) and work characteristics (e.g., supervisor support, job stress, and discrimination) did not account for the benefits of having supportive coworkers helps solidify coworker support as an important contributor to the well-being of workers. Indeed, supportive coworkers had a generally enriching effect and predicted outcomes in conceptually logical ways. Specifically, coworker support prevented the spill-over of stressful workplace conditions to an individual’s home environment and did not consistently predict the extent to which family environments impact work (presumably because the source of family conflict is often not a person’s coworkers). The fact that similar associations were found among both older (Study 1) and younger adults (Study 2) demonstrates that many of the dynamics leading to work and family interference apply to different developmental periods. Although Study 2 was much smaller in size and scope, the elucidation of burnout being the primary reason why positive work environments (resulting from positive coworker relationships) affect work/life conflict was an important contribution.

However, there are also limitations that are worth acknowledging. First, Study 1 was cross-sectional. Study 2 was lagged, although we had only a 2-month waiting period between data collections. Thus, in the absence of experimental and additional longitudinal data, it is difficult to draw causal conclusions about the processes examined in the current study. Longitudinal data could help in aiding in interpretation and causality but also seeing the rate at which work conditions interfere with family life and vice versa. Ultimately, more tightly controlled, longitudinal studies are needed to answer the time course and causal sensitivity of the model we proposed.

Second, although we examined the mediating effects of burnout and work environment on the association between coworker support and work-family outcomes, job satisfaction, and turnover, it is still unclear how each mediator can be improved on or cultivated (or minimized) in organizational settings. With multiple approaches for how burnout can be prevented – each focusing on specific professions and reporting varying degrees of success – the best burnout prevention techniques that are applicable to a wide range of occupations are still elusive (Greer and Greer, 1992; Simoni and Paterson, 1997; Espeland, 2006; Hätinen et al., 2009). Therefore, further research that includes a variety of prevention and reduction methods, as well as their application in a variety of professions, is needed to provide organizations with concrete and actionable steps to reduce burnout.

Third, another problem that must be addressed is the possibility that other factors (i.e., “third variables”) could also explain the effects of coworker support on work-family interference/enhancement, but these variables were not assessed. Thus, it would be beneficial and worthwhile to include and account for additional variables in the link between coworker support and work-life outcomes, such as class differences or other individual characteristics (e.g., perceived control, disability status, gender, union status; Gutek et al., 1991; Thomas and Ganster, 1995; Frone et al., 1992; Gillen et al., 2002; Chopik, 2015; Liu et al., 2015).

## Final Conclusion

In two studies, we found that coworker support was associated with better working environments, less intention to leave the organization (i.e., turnover) and burnout, increased job satisfaction, and more work-family and family-work enhancement. Work-family interference is a complex topic of discussion. With work demands consistently rising, it is important that researchers understand the factors that can reduce work-family and family-work interference, what increases work-family and family-work enhancement, and what mediates the effects of variables (e.g., coworker support) on these outcomes. Additional research will be helpful in further understanding the nuances of work-family interference to ultimately help organizations better their workers’ lives.

## Author’s Note

Portions of this work were presented as a poster at the Michigan State University’s Undergraduate Research and Arts Forum. The authors hold the copyright to this work.

## Data Availability Statement

The data from the Health and Retirement Study is publicly available for researchers. The study can be accessed via https://hrs.isr.umich.edu/.

## Ethics Statement

Because we analyzed an existing data source (for Study 1), the Michigan State Institutional Review Board considered this research exempt from ethical oversight as it did not constitute human subjects research (IRB# 17-1113). Data collection for Study 2 was approved by the Michigan State Institutional Review Board (IRB# x17-1182e). The patients/participants provided their written informed consent to participate in this study.

## Author Contributions

LN and WC conceived of the study. WC analyzed the data, tables, figures, and provided critical edits. LN drafted the initial manuscript.

## Conflict of Interest

The authors declare that the research was conducted in the absence of any commercial or financial relationships that could be construed as a potential conflict of interest.

## Footnotes

1 The use of self-reports is not without controversy. However, in many instances, self-reports can be useful assessments of individuals’ evaluations of their jobs, work/family intersections, and the support they receive from coworkers (if properly justified; Conway and Lance, 2010). In the current study, we were most interested in how people evaluated their jobs, the support they receive, and the ways in which work and family can conflict with or enhance one another. In this way, self-reports seemed like the most appropriate choice for the current studies. However, there are a series of studies demonstrating the convergent validity of these measures with other important, more objectively reported outcomes, such as turnover. Put simply, people unhappy with their jobs are more likely to quit. Likewise, perceiving support, over and above the actual support people receive, is related to higher well-being (Siedlecki et al., 2014). Given the large literature on all of the constructs used in the current study and objectively measured outcomes (e.g., by supervisors or independent observers), we were relatively comfortable with the use of self-reports in the current study.

2 We elected to examine coworker support specifically (instead of social support more broadly) for several reasons. First, we were interested in a work-specific form of support that might contribute to work environment and work-related burnout. It’s less clear how support from other sources (i.e., an individual’s family) would directly related to a job satisfaction or a workplace’s climate and environment. Second, and more practically, there were not parallel measures of social support that would make the contributions of these sources for employee well-being more comparable. However, we note that coworker support can fall under a more superordinate “social support” from which employees can benefit. Future researchers should directly integrate various types of social support in examining the effects of workplace environment on burnout and important job outcomes.

3 An alternative approach to modeling mediational processes via the PROCESS macro is to fit the model using structural equation modeling. Advantages of this alternate approach included the ability to model (a) measurement error of the constructs by estimating latent variables of each and (b) the covariances between the outcomes. This latter advantage is particularly useful because, in many cases, the outcome variables are intercorrelated. By accounting for these covariances, we can estimate the unique effects of each mediation model while accounting for the empirical overlap of all the outcomes. We conducted these analyses using MPlus (see Supplementary Material). The results can be found in Supplementary Tables S1–S3 (for Study 1) and 4–7 (for Study 2). The model fit for this approach revealed relatively good fit given the models’ complexity (Study 1: χ^2^ = 4502.64, *p* < 0.001; CFI = 0.940; RMSEA = 0.05; Study 2: χ^2^(1674) = 6089.44; CFI = 0.946; RMSEA = 0.07). The results of these analyses generally confirm those found in the text. Specifically, coworker support is associated with better work environments (and burnout in Study 2) which, in turn, are associated with better outcomes. The results from Study 1 were identical to those presented in text. For the exceptions, in some cases, after accounting for the covariation in the outcome variables, the ^∗^serial^∗^ indirect effects (coworker support > workplace environment > burnout > work/family enhancement) for the enhancement variables became not significant. However, the constituent mediations through work environment and burnout were still significant.

4 In Study 1, we found that many of these associations were present when participants completed all these questions in one setting. Because evaluative responses are often highly dependent on the mindset that an individual is adopting at any given time, we sought to collect data at a future time point to ensure that these relationships were robust and not a product of in-the-moment cognitive or evaluative biases in responding (Schwarz and Clore, 1983; Schwarz, 1999; Schwarz and Strack, 1999).
