# Supplementary material for: The Association Between Coworker Support and Work-Family Interference: A Test of Work Environment and Burnout as Mediators
Source: Front Psychol. 2020 May 5;11:819. doi: 10.3389/fpsyg.2020.00819 (PMC7214758; doi:10.3389/fpsyg.2020.00819)
Supplement: Supplementary file 1 [file Data_Sheet_1.DOCX]

| Supplementary Table 1. Path Estimates for Figure 1 using SEM | | | |
| --- | --- | --- | --- |
| Path | a | b | c' |
| Work-Family Interference | .61** | -.33** | -.02 |
| Family-Work Interference | .61** | -.15** | -.05* |
| Work-Family Enhancement | .61** | .40** | .02 |
| Family-Work Enhancement | .61** | .25** | .10** |
| Job Satisfaction | .61** | .62** | .12** |
| *Note*. * *p* < .05 ** *p* < .01 |  |  |  |

| Supplementary Table 2. Covariances between Outcomes in Study 1 using SEM | | | | |
| --- | --- | --- | --- | --- |
|  | 1 | 2 | 3 | 4 |
| 1.) Work-Family Interference |  |  |  |  |
| 2.) Family-Work Interference | .32** |  |  |  |
| 3.) Work-Family Enhancement | -.45** | -.17** |  |  |
| 4.) Famly-Work Enhancement | -.26** | -.31** | .57** |  |
| 5.) Job Satisfaction | -.17** | -.09** | .23** | .15** |
| *Note*. * *p* < .05 ** *p* < .01 |  |  |  |  |

| Supplementary Table 3. Indirect Effect Point Estimates for Figure 1 using SEM | | | | | |
| --- | --- | --- | --- | --- | --- |
| Indirect effects  of X on Y | Y = WF Int | Y = FW Int | Y = WF Enh | Y = FW Enh | Y = Job Satisfaction |
| Ind Effect | -.20 | -.09 | .25 | .16 | .38 |
| Ind Boot SE | .01 | .01 | .01 | .01 | .01 |
| Ind BootLLCI | -.23 | -.12 | .22 | .13 | .35 |
| Ind BootULCI | -.18 | -.07 | .27 | .18 | .40 |
| *Note*. W=Work, F=Family, Int=Interference, Enh=Enhancement, Boot SE=standard error, BootLLCI: Bootstrapped lower confidence interval, BootULCI: Bootstrapped upper confidence interval. Effects with confidence intervals that do not include zero are statistically significant. | | | | | |

| Supplementary Table 4. Path Estimates for Figure 2 using SEM | | | | | | |
| --- | --- | --- | --- | --- | --- | --- |
| Path | a | b | c | d | e | f’ |
| Work-Family Interference | .71** | -.15* | .61** | -.32** | .01 | -.05 |
| Family-Work Interference | .71** | -.15* | .52** | -.32** | .24** | -.21** |
| Work-Family Enhancement | .71** | -.15* | -.43** | -.32** | .35** | -0.08 |
| Family-Work Enhancement | .71** | -.15* | -.43** | -.32** | -.05 | .22** |
| Job Satisfaction | .71** | -.15* | -.35** | -.32** | .58** | .001 |
| Turnover | .71** | -.15* | .29** | -.32** | -.39** | .06 |
|  |  |  |  |  |  |  |

| Supplementary Table 5. Covariances between Outcomes in Study 2 using SEM | | | | | |
| --- | --- | --- | --- | --- | --- |
|  | 1 | 2 | 3 | 4 | 5 |
| 1.) Work-Family Interference |  |  |  |  |  |
| 2.) Family-Work Interference | .51** |  |  |  |  |
| 3.) Work-Family Enhancement | -.24** | .08 |  |  |  |
| 4.) Family-Work Enhancement | -.11 | -.24** | .68** |  |  |
| 5.) Job Satisfaction | -.16* | -.01 | .46** | .31** |  |
| 6.) Turnover | .17** | .09 | -.14* | -.09 | -.49** |
| *Note*. * *p* < .05 ** *p* < .01 |  |  |  |  |  |

| Supplementary Table 6. Indirect Effect Point Estimates for Figure 2 using SEM | | | | | | |
| --- | --- | --- | --- | --- | --- | --- |
| Indirect effects of X on Y | Y = WF Int | Y = FW Int | Y = WF Enh | Y = FW Enh | Y = Job Satisfaction | Y = Turnover |
| Ind1 Effect | .01 | .17 | .25 | -.04 | .41 | -.28 |
| Ind1 Boot SE | .05 | .05 | .05 | .06 | .05 | .06 |
| Ind1 BootLLCI | -.09 | .08 | .15 | -.15 | .31 | -.38 |
| Ind1 BootULCI | .11 | .27 | .35 | .08 | .51 | -.17 |
| Ind2 Effect | -.06 | -.05 | .05 | .05 | .04 | -.03 |
| Ind2 Boot SE | .03 | .03 | .02 | .02 | .02 | .02 |
| Ind2 BootLLCI | -.12 | -.11 | -.001 | -.001 | .001 | -.06 |
| Ind2 BootULCI | -.002 | -.002 | .09 | .09 | .07 | .000 |
| Ind3 Effect | -.19 | -.16 | .14 | .14 | .11 | -.09 |
| Ind3 Boot SE | .04 | .04 | .03 | .03 | .03 | .03 |
| Ind3 BootLLCI | -.28 | -.24 | .07 | .08 | .06 | -.15 |
| Ind3 BootULCI | -.11 | -.09 | .20 | .20 | .17 | -.04 |
| *Note*. W=Work, F=Family, Int=Interference, Enh=Enhancement, Boot SE=standard error, BootLLCI: Bootstrapped lower confidence interval, BootULCI: Bootstrapped upper confidence interval. Ind1=a*e. Ind2=a*b*c. Ind3=d*c (see Figure 2). Effects with confidence intervals that do not include zero are statistically significant. | | | | | | |

| Supplementary Table 7. Fit statistics for the constructs used in Study 2. | | | |  |  |
| --- | --- | --- | --- | --- | --- |
|  | Chi-square statistic (df) | RMSEA | CFI | TLI | SRMR |
| Coworker Support | (27) 82.05 | .074 | .984 | .975 | .020 |
| Turnover | (3) 734.71 | .064 | .987 | .975 | .020 |
| Burnout | (77) 355.28 | .095 | .947 | .930 | .056 |
| Work/Family Interference (all four measures covarying) | (48) 260.62 | .098 | .956 | .940 | .035 |
| Job Satisfaction | (27) 136.95 | .098 | .969 | .956 | .025 |
| Work Environment | (65) 335.98 | .096 | .914 | .895 | .057 |

| Supplementary Table 8. Main Analyses for Study 1 with Additional Controls | | | | | | | |
| --- | --- | --- | --- | --- | --- | --- | --- |
| Work Environment | *b* | *SE* | *β* | *t* | *p* | 95% CI | |
| Intercept | 1.620 | 0.085 |  | 19.054 | < .001 | 1.453 | 1.787 |
| Coworker Support | 0.266 | 0.012 | 0.316 | 22.001 | < .001 | 0.242 | 0.289 |
| Gender | -0.052 | 0.013 | -0.049 | -4.001 | < .001 | -0.078 | -0.027 |
| Age | 0.003 | 0.001 | 0.041 | 3.365 | 0.001 | 0.001 | 0.004 |
| Education | -0.002 | 0.002 | -0.010 | -0.792 | 0.428 | -0.006 | 0.003 |
| Hours/week | -0.001 | 0.001 | -0.016 | -1.324 | 0.186 | -0.002 | 0.000 |
| Tenure | 0.002 | 0.001 | 0.034 | 2.931 | 0.003 | 0.001 | 0.003 |
| Depression | -0.001 | 0.004 | -0.003 | -0.201 | 0.841 | -0.009 | 0.007 |
| Perceived Control | 0.022 | 0.007 | 0.042 | 3.325 | 0.001 | 0.009 | 0.035 |
| Extraversion | 0.043 | 0.014 | 0.045 | 3.066 | 0.002 | 0.016 | 0.071 |
| Agreeableness | -0.006 | 0.016 | -0.006 | -0.397 | 0.692 | -0.037 | 0.024 |
| Conscientiousness | 0.052 | 0.016 | 0.042 | 3.265 | 0.001 | 0.021 | 0.083 |
| Neuroticism | -0.023 | 0.011 | -0.026 | -2.003 | 0.045 | -0.045 | 0.000 |
| Openness to Experience | -0.002 | 0.014 | -0.002 | -0.116 | 0.908 | -0.030 | 0.026 |
| Supervisor Support | 0.220 | 0.011 | 0.306 | 20.386 | < .001 | 0.199 | 0.241 |
| Optimism | 0.030 | 0.007 | 0.055 | 4.011 | < .001 | 0.015 | 0.044 |
| Workplace Discrimination | -0.108 | 0.008 | -0.193 | -14.392 | < .001 | -0.123 | -0.093 |
|  |  |  |  |  |  |  |  |
| Work Interfering w/Family | *b* | *SE* | *β* | *t* | *p* | 95% CI | |
| Intercept | 0.665 | 0.113 |  | 5.874 | < .001 | 0.443 | 0.887 |
| Coworker Support | -0.049 | 0.016 | -0.054 | -3.067 | 0.002 | -0.081 | -0.018 |
| Gender | 0.034 | 0.017 | 0.029 | 1.970 | 0.049 | < .001 | 0.068 |
| Age | 0.002 | 0.001 | 0.020 | 1.362 | 0.173 | -0.001 | 0.004 |
| Education | 0.020 | 0.003 | 0.093 | 6.341 | < .001 | 0.014 | 0.026 |
| Hours/week | 0.011 | 0.001 | 0.237 | 15.737 | < .001 | 0.009 | 0.012 |
| Tenure | -0.001 | 0.001 | -0.027 | -1.887 | 0.059 | -0.003 | 0.000 |
| Depression | 0.056 | 0.005 | 0.160 | 10.479 | < .001 | 0.046 | 0.067 |
| Perceived Control | -0.027 | 0.009 | -0.047 | -3.079 | 0.002 | -0.044 | -0.010 |
| Extraversion | -0.096 | 0.019 | -0.091 | -5.092 | < .001 | -0.133 | -0.059 |
| Agreeableness | -0.001 | 0.021 | -0.001 | -0.057 | 0.955 | -0.042 | 0.039 |
| Conscientiousness | -0.020 | 0.021 | -0.015 | -0.934 | 0.350 | -0.061 | 0.022 |
| Neuroticism | 0.109 | 0.015 | 0.116 | 7.202 | < .001 | 0.079 | 0.139 |
| Openness to Experience | 0.058 | 0.019 | 0.053 | 3.073 | 0.002 | 0.021 | 0.095 |
| Supervisor Support | -0.025 | 0.014 | -0.032 | -1.761 | 0.078 | -0.054 | 0.003 |
| Optimism | -0.021 | 0.010 | -0.035 | -2.105 | 0.035 | -0.040 | -0.001 |
| Workplace Discrimination | 0.158 | 0.010 | 0.260 | 15.867 | 0.000 | 0.139 | 0.178 |
|  |  |  |  |  |  |  |  |
| Family Interfering w/ Work | *b* | *SE* | *β* | *t* | *p* | 95% CI | |
| Intercept | 1.010 | 0.071 |  | 14.141 | < .001 | 0.870 | 1.150 |
| Coworker Support | -0.022 | 0.010 | -0.042 | -2.144 | 0.032 | -0.042 | -0.002 |
| Gender | 0.002 | 0.011 | 0.003 | 0.197 | 0.844 | -0.019 | 0.024 |
| Age | -0.001 | 0.001 | -0.017 | -1.020 | 0.308 | -0.002 | 0.001 |
| Education | 0.003 | 0.002 | 0.028 | 1.691 | 0.091 | -0.001 | 0.007 |
| Hours/week | 0.001 | 0.000 | 0.025 | 1.459 | 0.145 | < .001 | 0.001 |
| Tenure | 0.001 | 0.000 | 0.019 | 1.214 | 0.225 | < .001 | 0.001 |
| Depression | 0.028 | 0.003 | 0.143 | 8.384 | < .001 | 0.022 | 0.035 |
| Perceived Control | -0.010 | 0.006 | -0.030 | -1.784 | 0.074 | -0.021 | 0.001 |
| Extraversion | -0.008 | 0.012 | -0.013 | -0.644 | 0.520 | -0.031 | 0.016 |
| Agreeableness | 0.012 | 0.013 | 0.018 | 0.929 | 0.353 | -0.013 | 0.038 |
| Conscientiousness | -0.089 | 0.013 | -0.118 | -6.719 | < .001 | -0.115 | -0.063 |
| Neuroticism | 0.065 | 0.010 | 0.122 | 6.777 | < .001 | 0.046 | 0.083 |
| Openness to Experience | 0.030 | 0.012 | 0.049 | 2.537 | 0.011 | 0.007 | 0.054 |
| Supervisor Support | 0.023 | 0.009 | 0.052 | 2.550 | 0.011 | 0.005 | 0.041 |
| Optimism | -0.018 | 0.006 | -0.055 | -2.969 | 0.003 | -0.030 | -0.006 |
| Workplace Discrimination | 0.048 | 0.006 | 0.139 | 7.623 | < .001 | 0.036 | 0.060 |
|  |  |  |  |  |  |  |  |
| Work Enhancing Family | *b* | *SE* | *β* | *t* | *p* | 95% CI | |
| Intercept | 2.568 | 0.176 |  | 14.571 | < .001 | 2.223 | 2.914 |
| Coworker Support | 0.080 | 0.025 | 0.057 | 3.195 | 0.001 | 0.031 | 0.129 |
| Gender | -0.093 | 0.027 | -0.052 | -3.431 | 0.001 | -0.145 | -0.040 |
| Age | 0.009 | 0.002 | 0.076 | 4.928 | < .001 | 0.005 | 0.012 |
| Education | -0.012 | 0.005 | -0.038 | -2.506 | 0.012 | -0.022 | -0.003 |
| Hours/week | -0.012 | 0.001 | -0.173 | -11.219 | < .001 | -0.014 | -0.010 |
| Tenure | 0.001 | 0.001 | 0.009 | 0.609 | 0.543 | -0.002 | 0.003 |
| Depression | -0.061 | 0.008 | -0.115 | -7.339 | < .001 | -0.078 | -0.045 |
| Perceived Control | 0.054 | 0.014 | 0.062 | 3.947 | < .001 | 0.027 | 0.080 |
| Extraversion | 0.160 | 0.029 | 0.100 | 5.463 | < .001 | 0.103 | 0.217 |
| Agreeableness | 0.032 | 0.032 | 0.017 | 0.988 | 0.323 | -0.031 | 0.095 |
| Conscientiousness | 0.120 | 0.033 | 0.059 | 3.682 | < .001 | 0.056 | 0.185 |
| Neuroticism | -0.057 | 0.024 | -0.040 | -2.424 | 0.015 | -0.103 | -0.011 |
| Openness to Experience | -0.011 | 0.030 | -0.006 | -0.359 | 0.720 | -0.068 | 0.047 |
| Supervisor Support | 0.156 | 0.022 | 0.129 | 6.930 | < .001 | 0.112 | 0.200 |
| Optimism | 0.099 | 0.015 | 0.110 | 6.468 | < .001 | 0.069 | 0.129 |
| Workplace Discrimination | -0.119 | 0.016 | -0.128 | -7.660 | < .001 | -0.149 | -0.089 |
|  |  |  |  |  |  |  |  |
| Family Enhancing Work | *b* | *SE* | *β* | *t* | *p* | 95% CI | |
| Intercept | 2.752 | 0.167 |  | 16.443 | < .001 | 2.424 | 3.080 |
| Coworker Support | 0.156 | 0.024 | 0.122 | 6.562 | < .001 | 0.109 | 0.203 |
| Gender | -0.043 | 0.026 | -0.026 | -1.662 | 0.097 | -0.093 | 0.008 |
| Age | 0.005 | 0.002 | 0.047 | 2.980 | 0.003 | 0.002 | 0.008 |
| Education | -0.010 | 0.005 | -0.032 | -2.076 | 0.038 | -0.019 | -0.001 |
| Hours/week | -0.003 | 0.001 | -0.053 | -3.337 | 0.001 | -0.005 | -0.001 |
| Tenure | 0.000 | 0.001 | 0.003 | 0.179 | 0.858 | -0.002 | 0.002 |
| Depression | -0.072 | 0.008 | -0.147 | -9.056 | < .001 | -0.088 | -0.056 |
| Perceived Control | 0.080 | 0.013 | 0.100 | 6.154 | < .001 | 0.054 | 0.105 |
| Extraversion | 0.042 | 0.028 | 0.029 | 1.522 | 0.128 | -0.012 | 0.097 |
| Agreeableness | 0.073 | 0.031 | 0.043 | 2.384 | 0.017 | 0.013 | 0.133 |
| Conscientiousness | 0.196 | 0.031 | 0.105 | 6.305 | < .001 | 0.135 | 0.257 |
| Neuroticism | -0.089 | 0.022 | -0.068 | -3.967 | < .001 | -0.133 | -0.045 |
| Openness to Experience | -0.016 | 0.028 | -0.011 | -0.587 | 0.557 | -0.071 | 0.039 |
| Supervisor Support | 0.022 | 0.021 | 0.020 | 1.027 | 0.304 | -0.020 | 0.064 |
| Optimism | 0.134 | 0.015 | 0.163 | 9.238 | < .001 | 0.106 | 0.163 |
| Workplace Discrimination | -0.010 | 0.015 | -0.012 | -0.692 | 0.489 | -0.039 | 0.019 |
|  |  |  |  |  |  |  |  |
| Job Satisfaction | *b* | *SE* | *β* | *t* | *p* | 95% CI | |
| Intercept | 1.854 | 0.086 |  | 21.590 | < .001 | 1.685 | 2.022 |
| Coworker Support | 0.099 | 0.012 | 0.122 | 8.090 | < .001 | 0.075 | 0.123 |
| Gender | -0.015 | 0.013 | -0.015 | -1.141 | 0.254 | -0.041 | 0.011 |
| Age | 0.003 | 0.001 | 0.049 | 3.785 | < .001 | 0.002 | 0.005 |
| Education | 0.000 | 0.002 | -0.003 | -0.211 | 0.833 | -0.005 | 0.004 |
| Hours/week | 0.000 | 0.001 | 0.006 | 0.494 | 0.621 | -0.001 | 0.001 |
| Tenure | 0.003 | 0.001 | 0.061 | 4.991 | < .001 | 0.002 | 0.004 |
| Depression | -0.008 | 0.004 | -0.025 | -1.926 | 0.054 | -0.016 | 0.000 |
| Perceived Control | 0.031 | 0.007 | 0.062 | 4.712 | < .001 | 0.018 | 0.044 |
| Extraversion | 0.033 | 0.014 | 0.036 | 2.312 | 0.021 | 0.005 | 0.061 |
| Agreeableness | -0.023 | 0.016 | -0.021 | -1.454 | 0.146 | -0.054 | 0.008 |
| Conscientiousness | 0.037 | 0.016 | 0.031 | 2.296 | 0.022 | 0.005 | 0.068 |
| Neuroticism | -0.046 | 0.012 | -0.056 | -4.025 | < .001 | -0.069 | -0.024 |
| Openness to Experience | 0.011 | 0.014 | 0.012 | 0.788 | 0.430 | -0.017 | 0.040 |
| Supervisor Support | 0.252 | 0.011 | 0.363 | 23.121 | < .001 | 0.231 | 0.274 |
| Optimism | 0.048 | 0.007 | 0.091 | 6.381 | < .001 | 0.033 | 0.062 |
| Workplace Discrimination | -0.130 | 0.008 | -0.242 | -17.181 | < .001 | -0.145 | -0.115 |

| Supplementary Table 9. Indirect Effect Point Estimates for Figure 1 from Models with Additional Controls | | | | | |
| --- | --- | --- | --- | --- | --- |
|  | Y= WF Int | Y = FW Int | Y = WF Enh | Y = FW Enh | Y = Job Satisfaction |
| Effect | -0.05 | -0.01 | 0.10 | 0.06 | 0.11 |
| Boot SE | 0.01 | 0.004 | 0.01 | 0.01 | 0.01 |
| BootLLCI | -0.06 | -0.02 | 0.08 | 0.04 | 0.09 |
| BootULCI | -0.04 | 0.0002 | 0.13 | 0.08 | 0.12 |
| *Note*. Boot SE=Bootstrapped standard error, BootLLCI: Bootstrapped lower confidence interval, BootULCI: Bootstrapped upper confidence interval. Effects with confidence intervals that do not include zero are statistically significant. | | | | | |

| Supplementary Table 10. Main Analyses for Study 12 with Additional Controls | | | | | | | |
| --- | --- | --- | --- | --- | --- | --- | --- |
| Work Environment | *b* | *SE* | *β* | *t* | *p* | 95% CI | |
| Intercept | 1.240 | 0.215 |  | 5.764 | < .001 | 0.818 | 1.663 |
| Coworker Support | 0.299 | 0.024 | 0.431 | 12.600 | < .001 | 0.252 | 0.345 |
| Gender | 0.024 | 0.045 | 0.015 | 0.536 | 0.592 | -0.064 | 0.113 |
| Age | 0.002 | 0.002 | 0.025 | 0.813 | 0.416 | -0.003 | 0.006 |
| Education | -0.014 | 0.020 | -0.019 | -0.693 | 0.489 | -0.053 | 0.026 |
| Hours/week | 0.001 | 0.002 | 0.013 | 0.453 | 0.650 | -0.004 | 0.006 |
| Tenure | -0.004 | 0.005 | -0.021 | -0.689 | 0.491 | -0.014 | 0.007 |
| Supervisor Support | 0.396 | 0.035 | 0.385 | 11.302 | < .001 | 0.327 | 0.465 |
| Job Stress | -0.172 | 0.041 | -0.122 | -4.173 | < .001 | -0.253 | -0.091 |
|  |  |  |  |  |  |  |  |
| Work Interfering w/ Family | *b* | *SE* | *β* | *t* | *p* | 95% CI | |
| Intercept | 0.546 | 0.232 |  | 2.360 | 0.019 | 0.092 | 1.001 |
| Coworker Support | -0.065 | 0.026 | -0.107 | -2.565 | 0.011 | -0.116 | -0.015 |
| Gender | -0.014 | 0.049 | -0.010 | -0.294 | 0.769 | -0.110 | 0.081 |
| Age | -0.003 | 0.002 | -0.049 | -1.287 | 0.199 | -0.008 | 0.002 |
| Education | 0.043 | 0.022 | 0.067 | 1.986 | 0.047 | < .001 | 0.085 |
| Hours/week | 0.001 | 0.003 | 0.015 | 0.446 | 0.656 | -0.004 | 0.006 |
| Tenure | -0.002 | 0.006 | -0.012 | -0.331 | 0.740 | -0.013 | 0.010 |
| Supervisor Support | -0.042 | 0.038 | -0.046 | -1.106 | 0.269 | -0.116 | 0.032 |
| Job Stress | 0.719 | 0.044 | 0.577 | 16.236 | < .001 | 0.632 | 0.806 |
|  |  |  |  |  |  |  |  |
| Family Interfering w/ Work | *b* | *SE* | *β* | *t* | *p* | 95% CI | |
| Intercept | 0.713 | 0.236 |  | 3.022 | 0.003 | 0.249 | 1.176 |
| Coworker Support | -0.059 | 0.026 | -0.106 | -2.264 | 0.024 | -0.110 | -0.008 |
| Gender | 0.171 | 0.049 | 0.133 | 3.459 | 0.001 | 0.074 | 0.268 |
| Age | -0.011 | 0.002 | -0.202 | -4.715 | < .001 | -0.016 | -0.007 |
| Education | 0.015 | 0.022 | 0.026 | 0.691 | 0.490 | -0.028 | 0.058 |
| Hours/week | < .001 | 0.003 | < .001 | -0.006 | 0.996 | -0.005 | 0.005 |
| Tenure | 0.017 | 0.006 | 0.122 | 2.878 | 0.004 | 0.005 | 0.029 |
| Supervisor Support | 0.054 | 0.038 | 0.066 | 1.410 | 0.159 | -0.021 | 0.130 |
| Job Stress | 0.444 | 0.045 | 0.394 | 9.837 | < .001 | 0.355 | 0.532 |
|  |  |  |  |  |  |  |  |
| Work Enhancing Family | *b* | *SE* | *β* | *t* | *p* | 95% CI | |
| Intercept | 2.178 | 0.286 |  | 7.629 | < .001 | 1.617 | 2.739 |
| Coworker Support | 0.107 | 0.031 | 0.153 | 3.393 | 0.001 | 0.045 | 0.169 |
| Gender | 0.147 | 0.060 | 0.091 | 2.448 | 0.015 | 0.029 | 0.264 |
| Age | 0.001 | 0.003 | 0.009 | 0.213 | 0.831 | -0.005 | 0.006 |
| Education | -0.016 | 0.027 | -0.023 | -0.618 | 0.537 | -0.069 | 0.036 |
| Hours/week | -0.007 | 0.003 | -0.075 | -1.997 | 0.046 | -0.013 | < .001 |
| Tenure | 0.023 | 0.007 | 0.133 | 3.266 | 0.001 | 0.009 | 0.038 |
| Supervisor Support | 0.217 | 0.047 | 0.209 | 4.671 | < .001 | 0.126 | 0.309 |
| Job Stress | -0.452 | 0.055 | -0.318 | -8.269 | < .001 | -0.559 | -0.344 |
|  |  |  |  |  |  |  |  |
| Family Enhancing Work | *b* | *SE* | *β* | *t* | *p* | 95% CI | |
| Intercept | 2.621 | 0.298 |  | 8.810 | < .001 | 2.037 | 3.205 |
| Coworker Support | 0.156 | 0.033 | 0.231 | 4.771 | < .001 | 0.092 | 0.221 |
| Gender | -0.136 | 0.062 | -0.087 | -2.185 | 0.029 | -0.259 | -0.014 |
| Age | 0.006 | 0.003 | 0.093 | 2.104 | 0.036 | < .001 | 0.012 |
| Education | -0.011 | 0.028 | -0.015 | -0.385 | 0.701 | -0.065 | 0.044 |
| Hours/week | 0.001 | 0.003 | 0.017 | 0.416 | 0.678 | -0.005 | 0.008 |
| Tenure | 0.011 | 0.007 | 0.066 | 1.518 | 0.130 | -0.003 | 0.026 |
| Supervisor Support | 0.003 | 0.048 | 0.003 | 0.058 | 0.954 | -0.092 | 0.098 |
| Job Stress | -0.352 | 0.057 | -0.256 | -6.193 | < .001 | -0.464 | -0.241 |
|  |  |  |  |  |  |  |  |
| Burnout | *b* | *SE* | *β* | *t* | *p* | 95% CI | |
| Intercept | 3.512 | 0.427 |  | 8.229 | < .001 | 2.673 | 4.350 |
| Coworker Support | -0.291 | 0.047 | -0.265 | -6.182 | < .001 | -0.383 | -0.198 |
| Gender | -0.225 | 0.090 | -0.089 | -2.512 | 0.012 | -0.401 | -0.049 |
| Age | -0.015 | 0.004 | -0.136 | -3.473 | 0.001 | -0.023 | -0.007 |
| Education | 0.020 | 0.040 | 0.017 | 0.500 | 0.618 | -0.058 | 0.098 |
| Hours/week | 0.007 | 0.005 | 0.051 | 1.428 | 0.154 | -0.003 | 0.017 |
| Tenure | -0.014 | 0.011 | -0.051 | -1.320 | 0.187 | -0.035 | 0.007 |
| Supervisor Support | -0.092 | 0.069 | -0.056 | -1.318 | 0.188 | -0.228 | 0.045 |
| Job Stress | 0.884 | 0.082 | 0.397 | 10.833 | < .001 | 0.724 | 1.044 |
|  |  |  |  |  |  |  |  |
| Job Satisfaction | *b* | *SE* | *β* | *t* | *p* | 95% CI | |
| Intercept | 2.103 | 0.158 |  | 13.296 | < .001 | 1.792 | 2.414 |
| Coworker Support | 0.111 | 0.017 | 0.240 | 6.394 | < .001 | 0.077 | 0.146 |
| Gender | 0.066 | 0.033 | 0.062 | 1.992 | 0.047 | 0.001 | 0.131 |
| Age | -0.003 | 0.002 | -0.066 | -1.911 | 0.057 | -0.006 | < .001 |
| Education | -0.010 | 0.015 | -0.020 | -0.649 | 0.517 | -0.039 | 0.019 |
| Hours/week | 0.001 | 0.002 | 0.024 | 0.771 | 0.441 | -0.002 | 0.005 |
| Tenure | 0.011 | 0.004 | 0.098 | 2.866 | 0.004 | 0.004 | 0.019 |
| Supervisor Support | 0.234 | 0.026 | 0.340 | 9.090 | < .001 | 0.184 | 0.285 |
| Job Stress | -0.325 | 0.030 | -0.345 | -10.741 | < .001 | -0.384 | -0.265 |
|  |  |  |  |  |  |  |  |
| Turnover | *b* | *SE* | *β* | *t* | *p* | 95% CI | |
| Intercept | 3.724 | 0.459 |  | 8.117 | < .001 | 2.822 | 4.625 |
| Coworker Support | -0.168 | 0.051 | -0.148 | -3.327 | 0.001 | -0.268 | -0.069 |
| Gender | -0.009 | 0.096 | -0.003 | -0.095 | 0.924 | -0.198 | 0.180 |
| Age | -0.007 | 0.005 | -0.058 | -1.432 | 0.153 | -0.016 | 0.002 |
| Education | 0.105 | 0.043 | 0.089 | 2.450 | 0.015 | 0.021 | 0.189 |
| Hours/week | -0.017 | 0.005 | -0.118 | -3.191 | 0.002 | -0.027 | -0.006 |
| Tenure | -0.032 | 0.012 | -0.111 | -2.763 | 0.006 | -0.055 | -0.009 |
| Supervisor Support | -0.389 | 0.075 | -0.231 | -5.210 | < .001 | -0.536 | -0.243 |
| Job Stress | 0.712 | 0.088 | 0.309 | 8.115 | < .001 | 0.540 | 0.884 |

| Supplementary Table 11. Indirect Effect Point Estimates for Figure 2 from Models with Additional Controls | | | | | | |
| --- | --- | --- | --- | --- | --- | --- |
| Indirect effects of X on Y | Y = WF Int | Y = FW Int | Y = WF Enh | Y = FW Enh | Y = Job Satisfaction | Y = Turnover |
| Ind1 Effect | -0.004 | 0.02 | 0.05 | -0.005 | 0.06 | -0.13 |
| Ind1 Boot SE | 0.01 | 0.01 | 0.02 | 0.02 | 0.01 | 0.03 |
| Ind1 BootLLCI | -0.03 | -0.005 | 0.02 | -0.04 | 0.04 | -0.21 |
| Ind1 BootULCI | 0.03 | 0.05 | 0.09 | 0.03 | 0.09 | -0.07 |
| Ind2 Effect | -0.05 | -0.05 | 0.04 | 0.05 | 0.03 | -0.05 |
| Ind2 Boot SE | 0.01 | 0.01 | 0.01 | 0.01 | 0.01 | 0.02 |
| Ind2 BootLLCI | -0.07 | -0.08 | 0.02 | 0.03 | 0.01 | -0.09 |
| Ind2 BootULCI | -0.03 | -0.03 | 0.07 | 0.08 | 0.04 | -0.02 |
| Ind3 Effect | -0.01 | -0.01 | 0.01 | 0.01 | 0.01 | -0.01 |
| Ind3 Boot SE | 0.01 | 0.01 | 0.005 | 0.01 | 0.003 | 0.01 |
| Ind3 BootLLCI | -0.02 | -0.02 | -0.001 | -0.003 | -0.0002 | -0.03 |
| Ind3 BootULCI | 0.001 | 0.001 | 0.02 | 0.02 | 0.01 | 0.001 |
| *Note*. W=Work, F=Family, Int=Interference, Enh=Enhancement, Boot SE=standard error, BootLLCI: Bootstrapped lower confidence interval, BootULCI: Bootstrapped upper confidence interval. Ind1=a*e. Ind2=a*b*c. Ind3=d*c (see Figure 2). Effects with confidence intervals that do not include zero are statistically significant. | | | | | | |
